# Peptide-Mediated Liposome Fusion: The Effect of Anchor Positioning

**DOI:** 10.3390/ijms19010211

**Published:** 2018-01-10

**Authors:** Niek S. A. Crone, Dirk Minnee, Alexander Kros, Aimee L. Boyle

**Affiliations:** Supramolecular and Biomaterials Chemistry, Leiden Institute of Chemistry, Gorlaeus Laboratories, Leiden University, Einsteinweg 55, 2333 CC Leiden, The Netherlands; n.s.a.crone@lic.leidenuniv.nl (N.S.A.C.); cdminnee@gmail.com (D.M.); a.kros@chem.leidenuniv.nl (A.K.)

**Keywords:** coiled coil, membrane fusion, cholesterol, membrane anchor, lipid

## Abstract

A minimal model system for membrane fusion, comprising two complementary peptides dubbed “E” and “K” joined to a cholesterol anchor via a polyethyleneglycol spacer, has previously been developed in our group. This system promotes the fusion of large unilamellar vesicles and facilitates liposome-cell fusion both in vitro and in vivo. Whilst several aspects of the system have previously been investigated to provide an insight as to how fusion is facilitated, anchor positioning has not yet been considered. In this study, the effects of placing the anchor at either the N-terminus or in the center of the peptide are investigated using a combination of circular dichroism spectroscopy, dynamic light scattering, and fluorescence assays. It was discovered that anchoring the “K” peptide in the center of the sequence had no effect on its structure, its ability to interact with membranes, or its ability to promote fusion, whereas anchoring the ‘E’ peptide in the middle of the sequence dramatically decreases fusion efficiency. We postulate that anchoring the ‘E’ peptide in the middle of the sequence disrupts its ability to form homodimers with peptides on the same membrane, leading to aggregation and content leakage.

## 1. Introduction

Within cells, the process of membrane fusion regulates a range of vital processes, including intracellular transport and exocytosis. In eukaryotic cells, these fusion events are regulated by a family of proteins named soluble *N*-ethylmaleimide-sensitive factor (NSF) attachment protein receptor (SNARE) proteins [1,2,3,4].

One specific SNARE-mediated membrane fusion process that has been widely studied in recent years is that of neurotransmitter release; neurotransmitters are packaged into vesicles functionalized with one SNARE protein and this interacts with two other SNARE proteins associated with the axonal presynaptic membrane to release neurotransmitters into the synaptic cleft [5,6]. This fusion process has four main steps: initially, the vesicle and axonal membranes are bought into close proximity by the three SNARE proteins from both membranes interacting to form a tetrameric coiled-coil; secondly, localized disruption of the membranes occurs; in the third step, the lipids of the outer membranes mix in a process known as hemifusion; and finally, the inner membranes mix and the contents are transferred [7].

Using the natural SNARE proteins to study membrane fusion processes is clearly the most accurate way to understand more about these mechanisms, but, due to the number of proteins involved, it is often difficult to isolate the roles of specific proteins [8], and some studies involving reconstituted SNAREs have failed to lead to fusion [9]. Developing model systems for membrane fusion may therefore be a solution to some of these issues, and in addition to providing fundamental mechanistic insights, model systems may also have applications as, for example, drug delivery systems. Numerous model systems have been developed in recent years with a range of molecules, such as deoxyribonucleic acid (DNA) [10,11,12,13], peptide nucleic acid (PNA) [14,15], peptides [16,17,18,19], and other small-molecule recognition complexes being employed as fusogens [20,21,22,23].

**Scheme 1:**
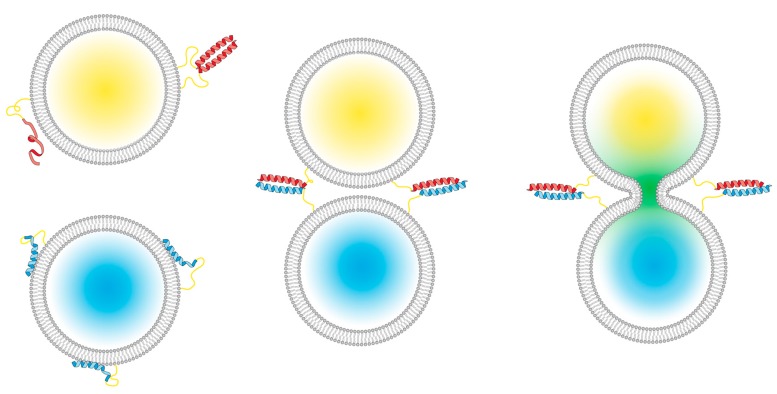
A peptide-mediated model system for membrane fusion. (**Left**): E (red) and K (blue) peptides are incorporated into liposomes; the E peptides are either unfolded or form homodimers, and the K peptides interact with lipid membranes [31]. Upon mixing the two liposome populations, (**middle**), coiled-coil heterodimers are formed between the two peptides; this brings the membranes into close proximity, which facilitates full fusion, also known as content mixing (**right**).

In our lab, we have developed a model system for membrane fusion which comprises two complementary coiled-coil-forming peptides known as peptide E and peptide K. These peptides are named due the prevalence of glutamic acid (E) residues in peptide E (EIAALEK)_n_ and lysines (K) in peptide K (KIAALKE)_n_ [24,25]. These peptides are linked to a cholesterol anchor via a polyethylene glycol (PEG) spacer, generating lipidated constructs dubbed CPE and CPK. This cholesterol anchors the peptides into liposomal membranes, and it has been previously demonstrated that, when CPE- and CPK-functionalized liposome populations are mixed, efficient and leakage-free lipid- and content-mixing occurs (Scheme 1) [18]. We have previously probed numerous aspects of this system, including the peptide length and orientation [26,27], the PEG length [28], and the anchor type [29]. Another study examined the effect of placing the anchor at the C- or N-terminus of the peptide or at both ends to generate a doubly anchored construct. It was discovered that the end at which the peptide was anchored, or indeed the presence of two anchors, did not affect either the rate or the extent of fusion [30].

In this study, we aim to probe the question of anchor positioning further by examining the effect of placing the anchor in the center of the peptide. This is of interest as the majority of SNARE proteins are held in membranes by hydrophobic transmembrane domains, but some SNARE proteins, such an SNAP-25, are tethered to the membrane via palmitoyl side-chains that are bound to cysteine residues found in the center of the protein sequence [32,33]. It would therefore be interesting to modify the E and K peptides so that the anchor is found at the center of the sequence and to compare these constructs to those which are anchored via the N-termini to determine which is the more effective anchor position.

This system was probed using a combination of circular dichroism (CD) spectroscopy, lipid- and content-mixing fluorescence assays, tryptophan fluorescence studies, and dynamic light scattering (DLS) to determine the effects of anchor positioning on peptide structure and both the rate and extent of membrane fusion.

It was discovered that placing the anchor in the center of the peptide sequence did not have a large effect on the structure of peptide K, but decreased the helicity of peptide E, although both peptides retained the ability to form a coiled-coil upon addition of the complementary peptide. Fusion was not affected when peptide K was anchored centrally, but strongly reduced lipid- and content-mixing was observed when the E peptide was anchored in this position. An increase in content leakage and a change in liposome size over time was also observed with this construct. It is postulated that anchoring the E peptide in the center of the sequence prevents homodimer formation with other peptides on the same liposome, leading to aggregation as homodimers are formed with peptides on neighboring liposomes. This aggregation then causes the liposomes to become unstable and limits the ability of this system to effectively facilitate lipid- and content-mixing.

## 2. Results

### 2.1. Design of the Study

**Figure 1:**
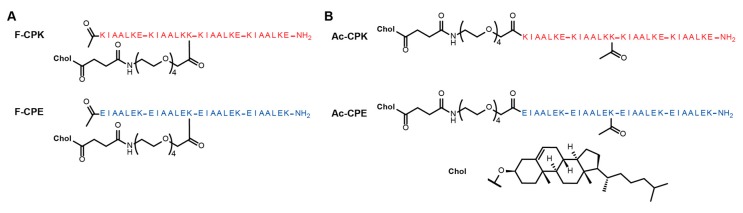
Structures of the cholesterol-anchored peptide constructs used in this study. (**A**) The structures of fCPK (top) and fCPE (bottom); (**B**) the structures of AcCPK (top) and AcCPE (bottom).

Inspired by the way in which some SNARE proteins, such as SNAP-25, are anchored to membranes [32,33], we decided to modify our model system to generate peptide constructs which had lipid anchors in the center of the peptide sequences. To facilitate this, the glutamic acid found at the ‘f’ position of the second heptad of the K peptide was mutated to lysine (Figure 1). A lysine was already found at this position in the E peptide, so no sequence modification was necessary. To enable anchor attachment, the highly acid labile methoxytrityl (mtt) protecting group was used to protect the lysine at this position. Upon completion of the peptide synthesis, this mtt group could be selectively deprotected to allow the PEG linker and cholesteryl anchor to be attached to the terminal amine group of this lysine side-chain, generating constructs named fCPE and fCPK, to reflect the position of anchor attachment (Figure 1A). The consequence of anchoring the peptide at this position is an increased overall charge, from +4 to +5 for the K peptide and from −4 to −5 for the E peptide. To test whether this increased charge would affect fusion, two control peptide constructs were synthesized which also possessed mtt-protected lysine in this position, but in this case the lysine side-chain was acetylated after synthesis to produce the control constructs AcCPE and AcCPK (Figure 1B), which possessed the same charge as the fCPE and fCPK peptides.

### 2.2. CD Spectroscopy

All four constructs were incorporated into liposomes and their secondary structures probed using CD spectroscopy. It is known that tethering peptides to a lipid surface can influence their secondary structure, particularly in the case of peptide K, which is required to interact with liposomes in order to facilitate fusion [31,34].

**Figure 2:**
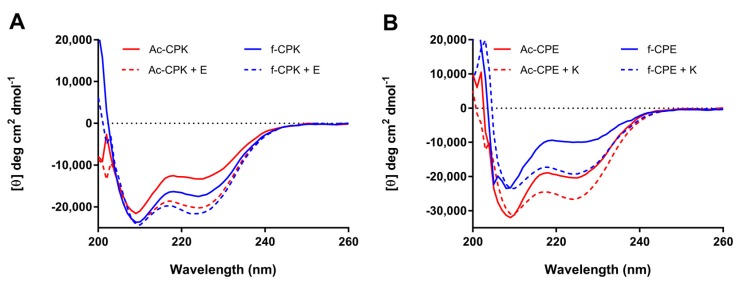
Circular dichroism (CD) spectra of the peptide constructs incorporated into liposomes. (**A**) AcCPK (red solid line) and fCPK (blue solid line) show comparable partial helicity when incorporated into liposomes, and upon addition of E, coiled-coil species are observed with both AcCPK (red dotted line) and fCPK (blue dotted line); (**B**) AcCPE (red solid line) and fCPE (blue solid line) are largely unfolded when incorporated into liposomes, but form coiled-coils upon K addition (red and blue dotted lines). (Total lipid) = 0.25 mM with 1 mol % cholesterol-anchored peptide and one equivalent E or K where stated, phosphate buffered saline (PBS), pH 7.4.

As is evident from Figure 2A, both fCPK and AcCPK exhibit similar CD spectra, which show a partial helical character when tethered to liposomes; this is consistent with previous observations for CPK [31]. Upon adding an equimolar amount of the complementary E peptide, the peak at 222 nm intensifies and the ratio between 208/222 nm is close to 1 (Table 1), which is indicative of coiled-coil formation. The differences in spectral shape and intensity between the two samples (fCPK + E and AcCPK + E) are negligible, indicating that anchor position does not affect the ability of the K peptide to form a coiled-coil with the E peptide.

In contrast, the spectra for fCPE and AcCPE, while similar in shape, show significant differences in intensity (Figure 2B). These spectra indicate that these peptides are also partially folded as *α*-helices, and previous work reveals that this partial helicity originates from homomeric interactions between the E-peptides on the liposome surface [28,31,34]. These spectra may therefore indicate that the ability of the fCPE peptide to form these homomeric interactions is greatly reduced. Upon the addition of peptide K to both AcCPE and fCPE, more helical species are observed, indicating both peptides form heteromeric coiled-coils with peptide K.

Collectively, these data indicate that the anchor position does not affect the secondary structure of the K-variants, but that the ability of the E peptide to form homomeric interactions may be disrupted when the anchor is placed in a central position. Whilst some of these intensity differences may result from concentration errors, as these peptides do not contain chromophores, the differences are too large to arise solely from such errors. Importantly, these data show that all of the constructs are still able to form coiled-coils effectively when the complementary peptide is added, and the spectra are similar to those observed when E and K are examined in the absence of liposomes (Figure S1). As coiled-coil formation is critical for liposome fusion these data indicate that, structurally, all of these constructs should be capable of promoting fusion.

### 2.3. Lipid Mixing

To determine whether fCPE and fCPK were capable of promoting lipid-mixing, an intermediate step in the fusion process, a Förster resonance energy transfer (FRET) assay was performed. To facilitate this, 0.5 mol % of both 1,2-dioleoyl-*sn*-glycero-3-phosphoethanolamine-*N*-(lissamine rhodamine B sulfonyl) (DOPE-LR) and 1,2-dioleoyl-*sn*-glycero-3-phosphoethanolamine-*N*-(7-nitro-2,1,3-benzoxadiazol-4-yl) (DOPE-NBD) were incorporated into liposomes containing fCPK or AcCPK. When ~100 nm liposomes are formed, the distance between the NBD and LR fluorophores is sufficiently small that FRET occurs between the two. Upon lipid-mixing, this distance increases and FRET no longer occurs, so the emission of the NBD fluorophore can be measured and if an increase in the intensity of the emission occurs this is indicative of lipid-mixing.

**Figure 3:**
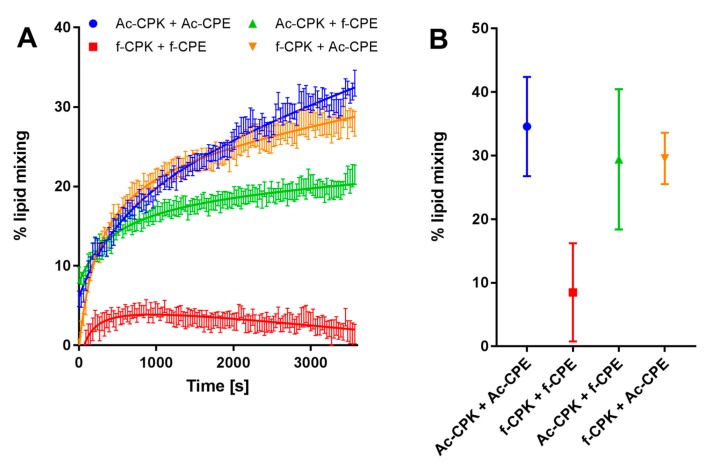
Lipid-mixing experiments for all combinations of fCPK, fCPE, AcCPK, and AcCPE. (**A**) Representative lipid-mixing plots for: AcCPK–AcCPE (blue); fCPK–fCPE (red); AcCPK–fCPE (green); and fCPK–AcCPE (orange). Error bars indicate the standard error of measurement between four samples. (**B**) Average and standard deviation of the total % of lipid-mixing for three independent sets of experiments with: AcCPK–AcCPE (blue); fCPK–fCPE (red); AcCPK–fCPE (green); and fCPK–AcCPE (orange). (Total lipid) = 100 µM of DOPC:DOPE:Cholesterol, 50:25:25 mol %, with 1 mol % cholesterol-anchored peptide, PBS, pH 7.4.

Figure 3A shows a representative experiment that monitors the increase in fluorescence intensity over 1 h for the fCPK and fCPE pairing, the control peptides, and mixed combinations. From this data, it can be concluded that the AcCPE and AcCPK pairing is most efficient at lipid-mixing, with 35% lipid-mixing being observed after one hour. If one of these peptides is swapped for the centrally anchored derivative, a small decrease in lipid-mixing is observed. This decrease is negligible for the AcCPE and fCPK pairing, whereas a slightly larger decrease is observed for the AcCPK-fCPE pairing. It should be noted, however, that the extent of lipid-mixing is highly variable for this pairing (Figure 3B). All three of these combinations exhibit comparable initial rates of lipid-mixing, indicating that substituting one of the N-terminally linked peptides for a centrally anchored variant has only a small effect on the efficiency of the hemifusion process.

When both fCPE and fCPK are employed, however, a significant drop in both the rate and extent of lipid-mixing is observed, meaning that only small amounts of lipid-mixing occur when both centrally-anchored peptide contructs are employed.

### 2.4. Content-Mixing

As lipid-mixing, or hemifusion, is an intermediate step in the full fusion (content-mixing) process, it is unlikely that the fCPE–fCPK pairing will efficiently promote content-mixing, as only low levels of lipid-mixing were observed, but this was still probed. Liposomes bearing fCPE or AcCPE were loaded with a self-quenching concentration of sulforhodamine B. If content-mixing with CPK-functionalised liposomes occurs, the sulforhodamine B will dilute below its self-quenching concentration and an increase in fluorescence will be observed.

**Figure 4:**
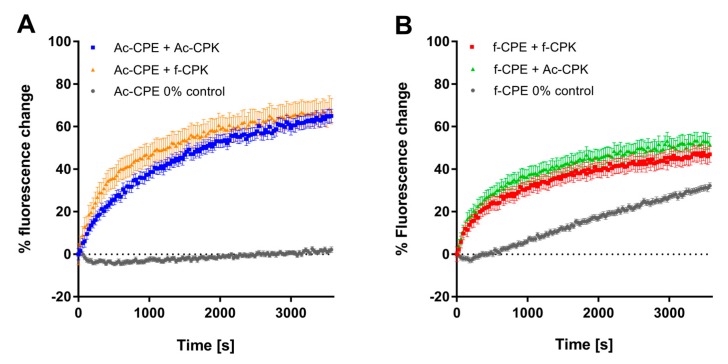
Content-mixing experiments for all combinations of fCPK, fCPE, AcCPK, and AcCPE. Representative content-mixing plots for: (**A**) AcCPE–AcCPK (blue) and AcCPE–fCPK (orange); (**B**) fCPE–fCPK (red) and fCPE–AcCPK (green). Zero percent controls represent samples of E-labeled liposomes mixed with plain liposomes. (Total lipid) = 100 µM of DOPC:DOPE:Cholesterol, 50:25:25 mol %, with 1 mol % cholesterol-anchored peptide, PBS, pH 7.4.

Figure 4 shows that the AcCPK–AcCPE pairing is capable of promoting content-mixing with no leakage (Figure S2). After one hour, 60% content-mixing is achieved, which is comparable to the amount of content-mixing facilitated by the CPE and CPK peptides used in previous studies [27,29]. The AcCPE–fCPK pairing also facilitates efficient content-mixing; both the rate and extent of mixing are comparable to the AcCPE–AcCPK pairing.

**Figure 5:**
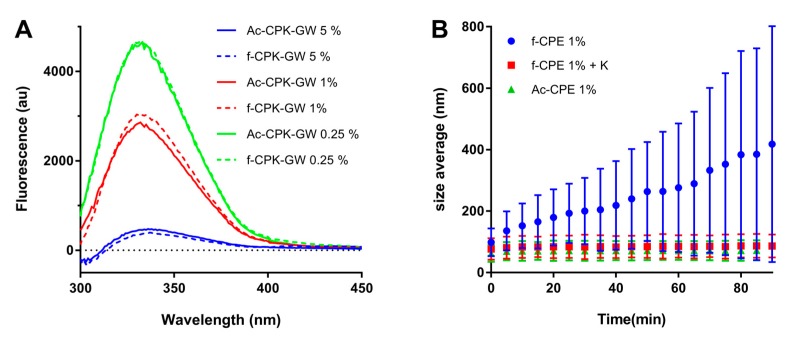
(**A**) Liposome-dependent tryptophan fluorescence spectra for fCPK peptide constructs (dotted lines) and AcCPK variants (solid lines) with a glycine and tryptophan on the C-terminus. Peptide concentration was kept constant at 2.5 µM, and the amount of lipids was varied to create liposomes with three different peptide fractions: 5% (blue), 1 % (red), and 0.25% (green). (**B**) Lipsome size increase monitored by dynamic light scattering (DLS). Size increase over 90 min for: fCPE-functionalized liposomes (blue); fCPE-functionalized liposomes with one equivalent of peptide K (red); and AcCPE-functionalized liposomes (green). Error bars indicate the 95% size distribution. (Total liposome) = 0.5 mM, PBS, pH 7.4.

The fact that both AcCPK and fCPK promote content-mixing when mixed with AcCPE is consistent with the CD data, which showed that both AcCPK and fCPK adopted partially helical structures when incorporated into liposomes and formed coiled-coils when the complementary E-peptide was added. This coiled-coil formation is the first step in the fusion process, and it is also important that the K-peptides are helical on liposomal membranes as their interaction with the liposome membranes is critical for fusion in this model system. To confirm that fCPK and AcCPK still interact with membranes, as the CD and content-mixing data suggest, variants bearing a tryptophan (trp) residue at their C-terminus were synthesized. Tryptophan fluorescence experiments were subsequently performed with these constructs at different peptide to lipid ratios, and these showed an increase in the intensity of the trp emission as the liposome concentration was increased (Figure 5A). A blue shift of the spectrum was also observed. Taken together, this fluorescence increase and the accompanying blue shift indicate that the trp residue is in a more hydrophobic environment when liposomes are present [36,37], suggesting that the peptide interacts with the liposomes. This has been observed for the original CPK variant [34,38], indicating that altering the charge of the peptide, for the AcCPK construct, coupled with changing the anchor position for fCPK, does not affect the ability of the peptide to interact with the liposome membrane.

When the fCPE construct is employed, it appears that content-mixing is observed for both the fCPE–fCPK and fCPE–AcCPK pairings, but when the 0% control is studied, a significant increase of fluorescence is also observed, indicating leakage of the dye from the fCPE liposomes (Figure 4B). The initial rate of leakage is different to the initial rate observed when fCPE is mixed with either fCPK or AcCPK, suggesting that some content-mixing may occur at first, but the final change in fluorescence for both samples with fCPE is 18% less than with the samples containing AcCPE. When leakage is accounted for (Figure S2), a relative fluorescence increase of only 17% for samples with fCPE, compared to 64% for samples with AcCPE, is observed. These figures suggest that a small amount of content-mixing seems to occur with the fCPE liposomes, but this fluorescence assay cannot differentiate adequately between content-mixing and leaking, therefore it is not possible to draw firm conclusions about whether content-mixing is occurring when the fCPE variant is employed.

### 2.5. Size Increase Studies

To probe this unusual behavior of fCPE further, dynamic light scattering (DLS) was employed to study the stability of the liposomes over time. Size changes of liposomes functionalized with both AcCPE and fCPE were investigated, and one equivalent of the complementary K peptide was added to fCPE-functionalized liposomes in another experiment. It is evident from Figure 5B that no significant size increase is observed for the AcCPE-functionalized liposomes, whereas the fCPE-functionalized liposomes increase in diameter from 100 nm to over 400 nm in 90 min. This change in size is accompanied by a significant change in the polydispersity of the sample, which is indicative of aggregate formation. When an equivalent of the K peptide is added to these liposomes, no size increase is observed over the same time period. Taken together, these data indicate that the size increase is likely to be due to homodimer formation between peptides anchored in different liposomes. The absence of a size increase when the K peptide is added is likely to be because EK heterodimers are formed, which are more stable than homodimers, and so the fCPE-functionalized liposomes are stabilized.

## 3. Discussion

We have demonstrated that changing the position of the cholesterol anchor site from the N-terminus to the center of the peptide affects E and K differently. CD spectroscopy demonstrates that both AcCPK and fCPK can interact with membranes; this is corroborated by an increase in trpfluorescence that is observed when trp-labelled derivatives of these constructs are mixed with liposomes. CD spectroscopy also revealed that both K constructs form coiled-coils when E is added. It has been demonstrated previously that both K-membrane interactions and EK-heterodimer formation are critical for effective fusion [28,34,38], so the fact that altering the anchor position has no effect suggests that these constructs should still be capable of promoting fusion. This is demonstrated to be the case, with both AcCPK and fCPK facilitating lipid- and content-mixing when combined with AcCPE.

Different behavior is observed for the AcCPE and fCPE derivatives. While AcCPE can promote leakage-free lipid- and content-mixing, reduced amounts of lipid-mixing are observed when the fCPE construct is employed, and significant leakage is observed during content-mixing when the fCPE construct is combined with either AcCPK or fCPK. An insight into what may be occurring is provided by the dynamic light scattering experiments, which show that the average particle size in a solution of fCPE-functionalized liposomes increases from 100 nm to 400 nm after 90 min. Previous studies of this system have shown that, in the absence of K, the E peptide exists primarily as a weakly associated homodimer on the surface of liposomes [31]. We postulate that, by anchoring the E peptide in the middle of the sequence, it is sterically prevented from forming homodimers with other peptides anchored in the same liposome. Homodimers therefore result from peptides on different liposomes interacting with each other, which leads to aggregation. This aggregation may subsequently lead to the destabilization of the liposomes, triggering content leakage, which is a phenomenon that has been observed in other peptide- and DNA-mediated fusion systems [16,39,40,41].

This hypothesis that f-position anchoring decreases homomeric interactions could also explain the significantly lower lipid-mixing for the fCPE–fCPK combination in comparison to fCPE–AcCPK. In our liposomal fusion model system, coiled-coil formation is the first step of the fusion process; this brings the membranes in close proximity but the peptides are still able to dissociate, which allows peptide K to interact with opposing liposomal membranes and promote complete fusion [38]. Changing the anchoring position may have a significant influence on the equilibrium between associated and dissociated E and K peptides, because there is a larger thermodynamic penalty for CPE to dissociate since it cannot form a stable homodimer. At the same time, changing the lipid attachment to form fCPK could disfavor the dissociation path where peptide K enters the membrane of the liposome that it is attached to, lowering the total amount of membrane-interacting peptide further.

Future studies could focus on probing this hypothesis further: AcCPE and fCPE constructs could be incorporated into the same liposome to determine whether homodimer formation is possible between the alternatively anchored constructs, and if so, this should decrease content leakage. FRET-pair labeled versions of CPE and CPK could be synthesized to observe the interactions of these peptides when two different populations of liposomes are mixed. Another possible explanation for the high levels of content leakage observed could be that the fCPE peptides are interacting with the liposomes. This is unlikely as the peptide sequence has not been significantly changed, but this could be probed by synthesizing tryptophan-functionalized derivatives and performing tryptophan fluorescence experiments in the absence and presence of liposomes.

## 4. Materials and Methods

### 4.1. Materials

Tentagel HL-RAM resin was obtained from Rapp Polymere (Tuebingen, Germany). Fmoc-protected amino acids and *O*-(1*H*-6-Cholorobenzotriazol-1-yl)-1,1,3,3-tetramethyluronium hexafluorophosphate (HCTU) were purchased from NovaBiochem (Amsterdam, The Netherlands). Acetic anhydride, acetonitrile, dimethylformamide (DMF), dioxane, methanol, piperidine, pyridine, and trifluoroacetic acid (TFA) were purchased from Biosolve (Valkenswaard, The Netherlands). *N*,*N*-Diisopropylethylamine (DIPEA) and Oxyma were purchased from Carl Roth (Karlsruhe, Germany). Cholesterol, cholesteryl hemisuccinate, Diisopropylcarbodiimide (DIC), *tert*-butanol, sulforhodamine B, sodium hydroxide, triisopropylsilane (TIPS), and trimethylphosphine (1 M in toluene) were obtained from Sigma Aldrich (Zwijndrecht, The Netherlands). Chloroform, dichloromethane (DCM), and diethyl ether were supplied by Honeywell (Meppel, The Netherlands). The compounds 1,2-dioleoyl-*sn*-glycero-3-phosphocholine (DOPC), 1,2-dioleoyl-*sn*-glycero-3-phosphoethanolamine (DOPE), 1,2-dioleoyl-*sn*-glycero-3-phosphoethanolamine-*N*-(lissamine rhodamine B sulfonyl) (DOPE-LR), and 1,2-dioleoyl-*sn*-glycero-3-phosphoethanolamine-*N*-(7-nitro-2,1,3-benzoxadiazol-4-yl) (DOPE-NBD) were all purchased from Avanti Polar Lipids (Alabaster, AL, USA). Ultrapure water was purified using a Milli-Q™ purification system from Millipore (Amsterdam, The Netherlands).

### 4.2. N_3_-PEG_4_-COOH Synthesis

Ethyl 14-Azido-3,6,9,12-tetraoxatetradecan-1-oate (N_3_-PEG_4_-COOEt) was synthesized according to literature methods [42]. N_3_-PEG_4_-COOEt (1.64 g, 5.4 mmol) was dissolved in MeOH (25 mL). NaOH (1 M, 8 mL) was added, and the reaction was stirred overnight or until thin-layer chromatography (TLC) indicated complete conversion of the starting material. The reaction was diluted with H_2_O (12 mL), and the methanol was evaporated. The aqueous layer was washed with DCM (25 mL), acidified with HCl, and extracted with DCM (3 × 25 mL). After drying with Na_2_SO_4_, the solvent was removed to yield the product as a viscous liquid in quantitative yield. ^1^H NMR (300 MHz, CDCl_3_) *δ* 10.80 (s, 1H), 4.07 (s, 2H), 3.64–3.63 (m, 2H), 3.6–3.55 (m, 12H), 3.28 (t, *J* = 6.0 Hz, 2H). ^13^C NMR (300 MHz, CDCl_3_) *δ* 173.73, 70.82, 70.35, 70.29, 70.23, 70.17, 69.75, 50.38, (Figure S4).

### 4.3. Peptide Synthesis

Peptide synthesis was performed using a microwave-assisted Liberty Blue automated synthesizer from CEM (Matthews, NC, USA). Peptides were synthesized on a 0.1 mmol scale using Tentagel HL-RAM resin with a loading capacity of 0.37 mmol g^−1^. Fmoc deprotection was achieved using a solution of 20% piperidine in DMF under heating at 90 °C for 1 min, followed by three washing steps. Coupling of amino acids was performed using 4 mL of coupling solution, containing 0.125 M of the respective amino acid, 0.25 M DIC as activator, and 0.125 M oxyma as base. Amino acid coupling was performed at 90 °C for 4 min.

To allow for selective deprotection and modification, mtt-protected lysine was used at position 14 of the peptide sequences. Once synthesis was complete, the peptide was first PEGylated or acylated on the N-terminus before modification of the mtt-protected lysine. The mtt-protecting group was selectively removed using DCM containing 3% TFA and 3% TIPS. Aliquots (2 mL) of this solution were added to the resin, which was shaken for 2 min before being washed with DMF. These steps were repeated until the yellow colour (indicative of the mtt-protecting group) was no longer observed. At this point, the peptides were PEGylated, reduced, lipidated, and cleaved as for the N-terminally anchored peptides.

Peptide PEGylation and lipidation was performed on resin, using two equivalents of the N_3_-PEG_4_-CH_2_-COOH spacer, HATU (two eq.) and DIPEA (four eq.). The resin was subsequently washed with DMF, and azide reduction was performed using trimethylphosphine (10 eq.) in 4:1 dioxane:water. After coupling, the resin was washed using 4:1 dioxane:water, DMF, and DCM. Lipidation was facilitated using cholesteryl hemisuccinate (two eq.) with HATU (two eq.) and DIPEA (four eq.). All conversions were confirmed using a Kaiser test [43]. The resin was washed with DMF and DCM before cleavage of the lipidated peptide construct from the resin was achieved using a 95:2.5:2.5 mixture of TFA:TIPS:water. The crude peptide was precipitated into cold diethyl ether, collected by centrifugation, redissolved in 20% acetonitrile/water, and lyophilized.

### 4.4. Peptide Purification

All peptides were purified by reversed-phase HPLC using a Shimadzu system comprising two LC-8A pumps and an SPD-10AVP UV-Vis detector. Lysine-rich peptides were purified on a Kinetix Evo C18 column, and Glutamic-acid-rich peptides were purified using a Vydac protein C4 column. Eluents used were water containing 0.1% TFA (A) and MeCN with 0.1% TFA (B); all peptides were eluted using a gradient of 20–90% B over 40 min at a flow rate of 15 mL min^−1^. The collected fractions were examined using LCMS, (Table S1 and Figures S5–S12), and the fractions containing purified peptide were pooled and freeze-dried.

### 4.5. Formation of Liposomes

One millimolar (1 mM) stock solutions containing DOPC:DOPE:cholesterol (50:25:25 mol %) or DOPC:DOPE:cholesterol:DOPE-LR:DOPE-NBD (49.5:24.75:24.75:0.5:0.5 mol %) were prepared in a 1:1 (*v*/*v*) chloroform:MeOH solution. Stock solutions of the lipidated peptides were prepared at 50 µM concentration in the same solvent system.

For the lipid-mixing assays, fluorescent liposomes containing K-lipopeptide constructs and non-fluorescent E-lipopeptide-containing liposomes were prepared at 500 µM concentration with 1 mol % lipopeptide. Lipid films were formed by evaporating the chloroform:MeOH solution under a stream of air and rehydrating the resulting film with PBS buffer. The solution was then sonicated at 55 °C for ~10 min to yield liposomes of ~100 nm diameter as assessed by dynamic light scattering (DLS) spectroscopy on a Zetasizer Nano S (Malvern Instruments, Malvern, UK) using PMMA low-volume cuvettes (VWR international, Leuven, Belgium) . After formulation, the solutions were diluted to the working concentration of 100 µM. Examples of size distributions of liposomes can be found in Figure S3.

For content-mixing assays, liposomes were prepared at 500 µM concentration with 1 mol % lipopeptide. Liposomes functionalized with K-lipopeptides were rehydrated with PBS and prepared in the same manner as liposomes used for lipid-mixing assays. Liposomes bearing E-lipopeptides were rehydrated with PBS containing 20 mM sulforhodamine B. The solution was then sonicated as before, and non-encapsulated sulforhodamine B was removed by passing the liposomes down a Sephadex G25 column (GE healthcare, Eindhoven, The Netherlands). After size exclusion, liposomes were diluted to the working concentration of 100 µM.

### 4.6. Fluorescence Spectroscopy

Lipid- and content-mixing assays were performed using a TECAN infinite M1000 PRO fluorimeter at 25 °C. All experiments were performed in 96-well plates.

For the lipid-mixing experiments, fluorescence intensity was measured continuously for 1 h by monitoring NBD emission at 530 nm after mixing equimolar amounts of E- and K-functionalised liposomes. The percentage of lipid-mixing between the liposomes was calculated according to Equation (1):

% lipid mixing = (*F*_(*t*)_ − *F*_0_)/(*F_max_* − *F*_0_) (1)

where *F*_(*t*)_ is the fluorescence intensity at time ‘*t*’, *F*_0_ is the baseline fluorescence value, which was determined by measuring the fluorescence intensity of NBD-containing, K-functionalised liposomes to which an equal amount of PBS had been added, and *F_max_* is the maximum fluorescence value, which was obtained by measuring the fluorescence intensity from liposomes containing half the concentration of fluorescent lipids (i.e., 0.25 mol % each of DOPE-LR and DOPE-NBD).

For content-mixing experiments, sulforhodamine B fluorescence intensity was measured for 60 min at 580 nm after mixing non-fluorescent K-liposomes with sulforhodamine B-containing E-liposomes. Negative control liposomes were prepared from the E-liposomes mixed with an equal number of plain liposomes. The percentage fluorescence increase was calculated using Equation (2):

% *F* = (*F*_(*t*)_ − *F*_0_)/*F*_0_ (2)

where *F*_0_ is defined as the fluorescence of each sample directly after mixing of the liposomes.

### 4.7. Tryptophan Fluorescence Experiments

Lipid films for the liposomes used in tryptophan fluorescence studies were prepared at a constant peptide concentration of 2.5 µM with varying amounts of lipid stock (DOPC:DOPE:cholesterol 50:25:25 mol %, 1 M total concentration) to create samples with different lipid equivalents. Tryptophan was excited at 280 nm with a 10 nm band gap, and fluorescence emission between 300 and 450 nm was collected. Each sample was measured three times with a 1 min interval, and the three spectra were averaged.

### 4.8. Size Increase Experiments 

Change in liposome size and size distribution were followed by DLS. Samples were measured every 5 min for 90 min at a constant temperature of 25 °C. For every time point, 10 measurements of 6 s each were performed and their values averaged. Samples were prepared at 0.5 mM lipid concentration with 1% substituted by the respective lipopeptide. The non-lipidated binding partner, one equivalent, was added to the liposomes as a 100 µM solution.

### 4.9. CD Experiments

CD spectra were measured using a JASCO J-815 CD spectrometer fitted with a Peltier temperature controller set to 25 °C. Samples were measured in quartz cuvettes with a 5 mm path length. Spectra were recorded from 260 to 190 nm at 1 nm intervals with a 1 nm bandwidth. Mean residue molar ellipticity, (*θ*), (deg cm^2^ dmol.res^−1^) was calculated according to Equation (3):

(3) $$\left[\theta \right]=\frac{100\;\times \;{\left(\theta \right)}_{obs}}{c\;\times \;n\;\times \;l}$$

where (*θ*)*_obs_* is the observed ellipticity in mdeg, *c* is the peptide concentration in mM, *n* is the number of peptide bonds, and *l* is the path length of the cuvette in cm. Percentage helicity was calculated from the residue molar ellipticity at 222 nm using the following Equation (4):

*F_helix_* = ((*θ*)_222_ − (*θ*)_0_)/((*θ*)*_max_* − (*θ*)_0_) (4)

With (*θ*)*_max_* as the maximum theoretical mean residue elipticity, defined as (*θ*)*_max_* = (*θ*)*_∞_* (*n* − *x*)/*n* for an *n* residue helix and *x* an arbitrary number of amino acids assumed not to participate in helix formation (we use three in all calculations). (*θ*)*_∞_* is defined as the theoretical helicity of an infinite *α*-helix and is temperature-dependent, defined via (*θ*)*_∞_* = (−44,000 + 250*T*), with *T* being the temperature in °C. The minimum signal expected for a random peptide coil at 222 nm is given as (*θ*)_0_, and is also temperature-dependent via the following relationship (*θ*)_0_ *=* 2220 − 53T [35].

## Figures and Tables

**Table 1:** Molar ellipticities and helicity information for the peptides used in this study.

Peptides	(*θ*)_222nm_ (deg cm^2^ dmol.res^−1^)	% *α*-helix ^a^	(*θ*)_222nm_/(*θ*)_208nm_
f-CPE	−9786	30%	0.42
f-CPE + K	−18,705	55%	0.84
Ac-CPE	−19,986	58%	0.64
Ac-CPE + K	−26,167	75%	0.89
f-CPK	−17,112	50%	0.75
f-CPK + E	−21,600	63%	0.94
Ac-CPK	−13,017	39%	0.62
Ac-CPK + E	−19,940	58%	0.87

^a^ Percentage helicity was calculated from the molar ellipticity at 222 nm using previously reported methods [35].

## Supplementary Materials

Supplementary materials can be found at www.mdpi.com/1422-0067/19/1/211/s1.

## Abbreviations

CD	Circular dichroism
DCM	Dichloromethane
DIC	Diisopropylcarbodiimide
DIPEA	Diisopropylethylamine
DLS	Dynamic light scattering
DMF	Dimethylformamide
DNA	Deoxyribonucleic acid
DOPC	1,2-dioleoyl-*sn*-glycero-3-phosphocholine
DOPE	1,2-dioleoyl-*sn*-glycero-3-phosphoethanolamine
DOPE-LR	1,2-dioleoyl-*sn*-glycero-3-phosphoethanolamine-*N*-(lissamine rhodamine B sulfonyl)
DOPE-NBD	1,2-dioleoyl-*sn*-glycero-3-phosphoethanolamine-*N*-(7-nitro-2,1,3-benzoxadiazol-4-yl)
Fmoc	Fluorenylmethyloxycarbonyl
FRET	Fluorescence resonance energy transfer
HATU	1-[Bis(dimethylamino)methylene]-1*H*-1,2,3-triazolo[4,5-b]pyridinium 3-oxid hexafluorophosphate
HPLC	High performance liquid chromatography
LUV	Large unilamellar vesicle
MeCN	Acetonitrile
MeOH	Methanol
Mtt	Methoxytrityl
NMR	Nuclear magnetic resonance
PBS	Phosphate buffered saline
PEG	Polyethylene glycol
PNA	Peptide nucleic acid
PMMA	Poly(methyl methacrylate)
SEM	Standard error of measurement
TIPS	Triisopropylsilane
TFA	Trifluoroacetic acid
TLC	Thin-layer chromatography
Trp	Tryptophan
SNARE	soluble NSF (*N*-ethylmaleimide-sensitive factor) attachment protein receptor
UV-Vis	Ultra violet-visible
