# Supplementary material for: Peptide-Mediated Liposome Fusion: The Effect of Anchor Positioning
Source: Int J Mol Sci. 2018 Jan 10;19(1):211. doi: 10.3390/ijms19010211 (PMC5796160; doi:10.3390/ijms19010211)
Supplement: Supplementary file 1 [file ijms-19-00211-s001.pdf]

CD spectra

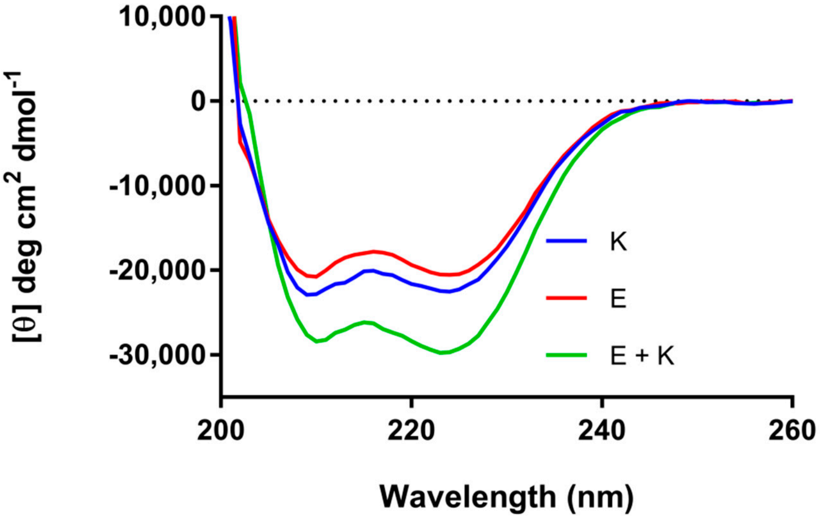

Figure S1: CD structures of the coiled-coil forming peptides E; $(EIAALEK)_4GW$ and K; $(KIAALKE)_4GW$ without liposomes. Conditions: [2.5 μM] peptide, PBS pH 7.4, 25 °C.

Content leaking

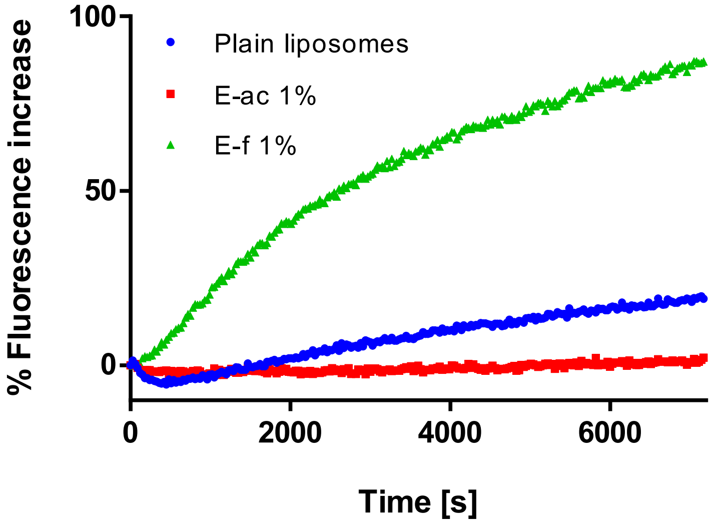

Figure S2: Leaking of sulforhodamine B from liposomes used in content mixing experiments. Liposomes [0.1 mM], comprise DOPC:DOPE:Cholesterol (50:25:25 mol%), with 20 mM Sulforhodamine B, (AcCPE, and fCPE functionalised liposomes), or 10 mM (plain liposomes) in PBS, pH 7.4.

Liposome Size Distributions

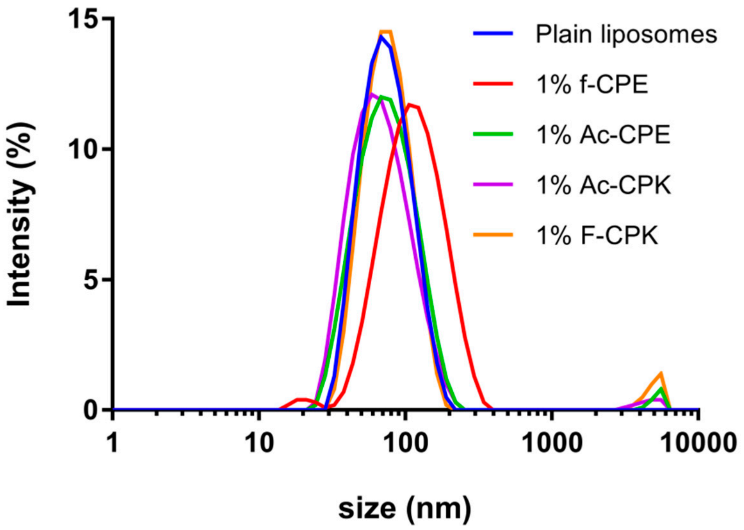

Figure S3: Size distribution by intensity of liposomes used in fusion experiments as measured using DLS. liposomes [0.5 mM], comprising DOPC:DOPE:Cholesterol (50:25:25 mol%) with 1% of the respective lipopeptide; f-CPE (Red), Ac-CPE (Green), f-CPK (yellow) and Ac-CPK (purple) in PBS at pH 7.4. Liposomes were subsequently diluted from 0.5 mM to the appropriate concentrations for CD, fluorescence, or lipid- and content-mixing experiments.

NMR spectra of N3-PEG4-COOH

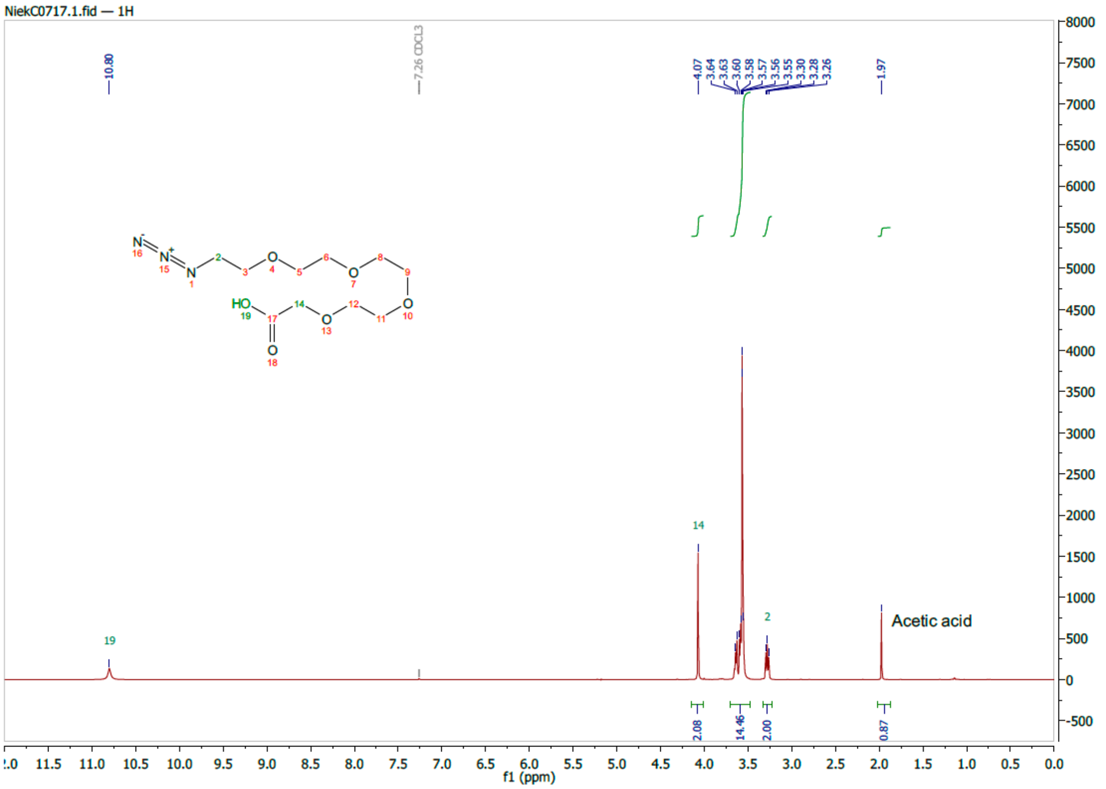

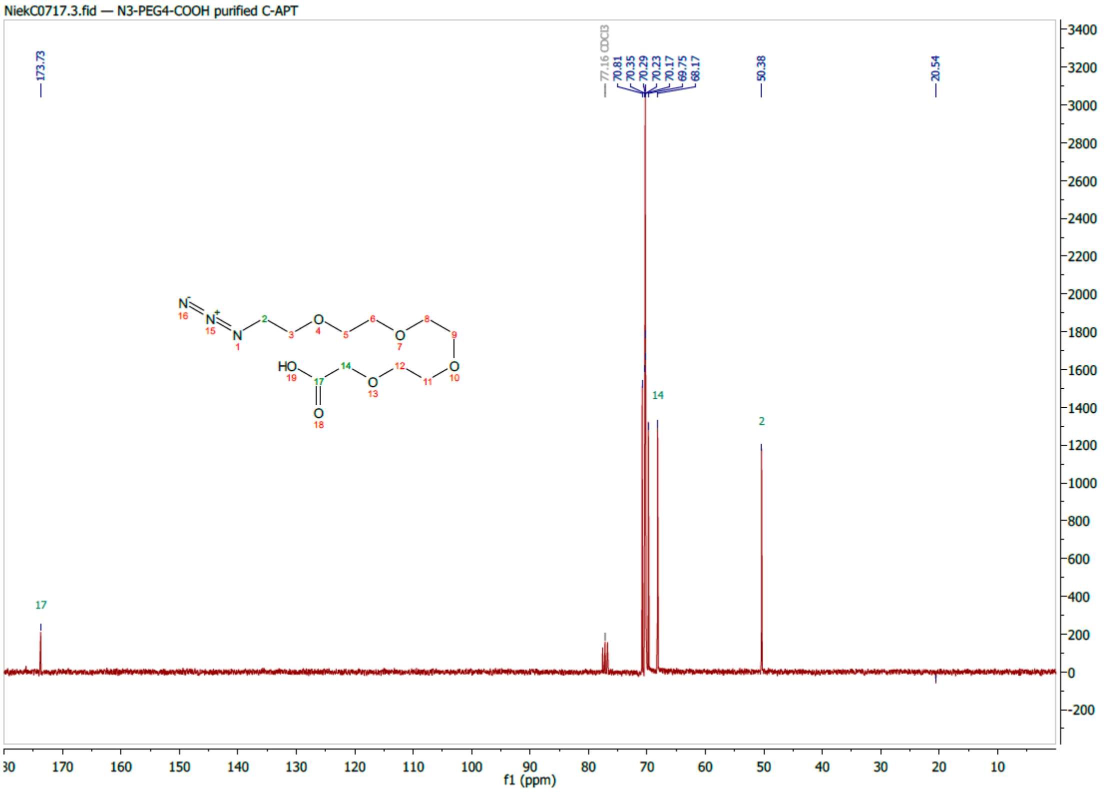

Figure S4: Proton (top) and carbon (bottom) NMR spectra of the N3-PEG4-COOH linker. Both spectra were recorded using $CDCl_3$ as solvent.

LC-MS of purified peptides

Peptide name	Calculated mass (Da)	Measured mass (Da)
AcCPE	$[M + 2H^+]^{2+}$ 1891.61<br>$[M + 2H^+\text{-cholesterol}]^{2+}$ 1706.85<br>$[M + 3H^+]^{3+}$ 1261.41	1890.21<br>1705.24<br>1260.77
fCPE	$[M + 2H^+]^{2+}$ 1891.61<br>$[M + 2H^+\text{-cholesterol}]^{2+}$ 1706.85<br>$[M + 3H^+]^{3+}$ 1261.41	1890,00<br>1705.94<br>1272.24
AcCPK	$[M + 2H^+]^{2+}$ 1889.74<br>$[M + 2H\text{-cholesterol}^+]^{2+}$ 1705.135<br>$[M + 3H^+]^{3+}$ 1260.16<br>$[M + 3H^+\text{-cholesterol}]^{3+}$ 1137.38	1887.90<br>1702.94<br>1257.96<br>1135.10
fCPK	$[M + 2H^+]^{2+}$ 1889.74<br>$[M + 2H\text{-cholesterol}^+]^{2+}$ 1705.135<br>$[M + 3H^+]^{3+}$ 1260.16<br>$[M + 3H^+\text{-cholesterol}]^{3+}$ 1137.38	1887.50<br>1703.36<br>1257.77<br>1134.50
AcCPK-GW	$[M + 2H^+]^{2+}$ 2010.29<br>$[M + 2H\text{-cholesterol}^+]^{2+}$ 1826.13<br>$[M + 3H^+]^{3+}$ 1340.52	2010.63<br>1824.69<br>1339.51
fCPK-GW	$[M + 2H^+]^{2+}$ 2010.29<br>$[M + 3H^+]^{3+}$ 1340.52	2010.14<br>1339.30
E_4GW	$[M + 2H^+]^{2+}$ 1661.41<br>$[M + 3H^+]^{3+}$ 1107.94	1660.33<br>1106.59
K_4GW	$[M + 2H^+ + 4\ TFA]^{2+}$ 1887.50<br>$[M + 2H^+ + 3\ TFA]^{2+}$ 1830.50<br>$[M + 3H^+ + 2\ TFA]^{3+}$ 1182.67<br>$[M + 3H^+ + TFA]^{3+}$ 1144.68	1886.13<br>1829.33<br>1182.27<br>1144.60

Table S1: Overview of the calculated masses of all peptides used in this project, and the masses found by LCMS.

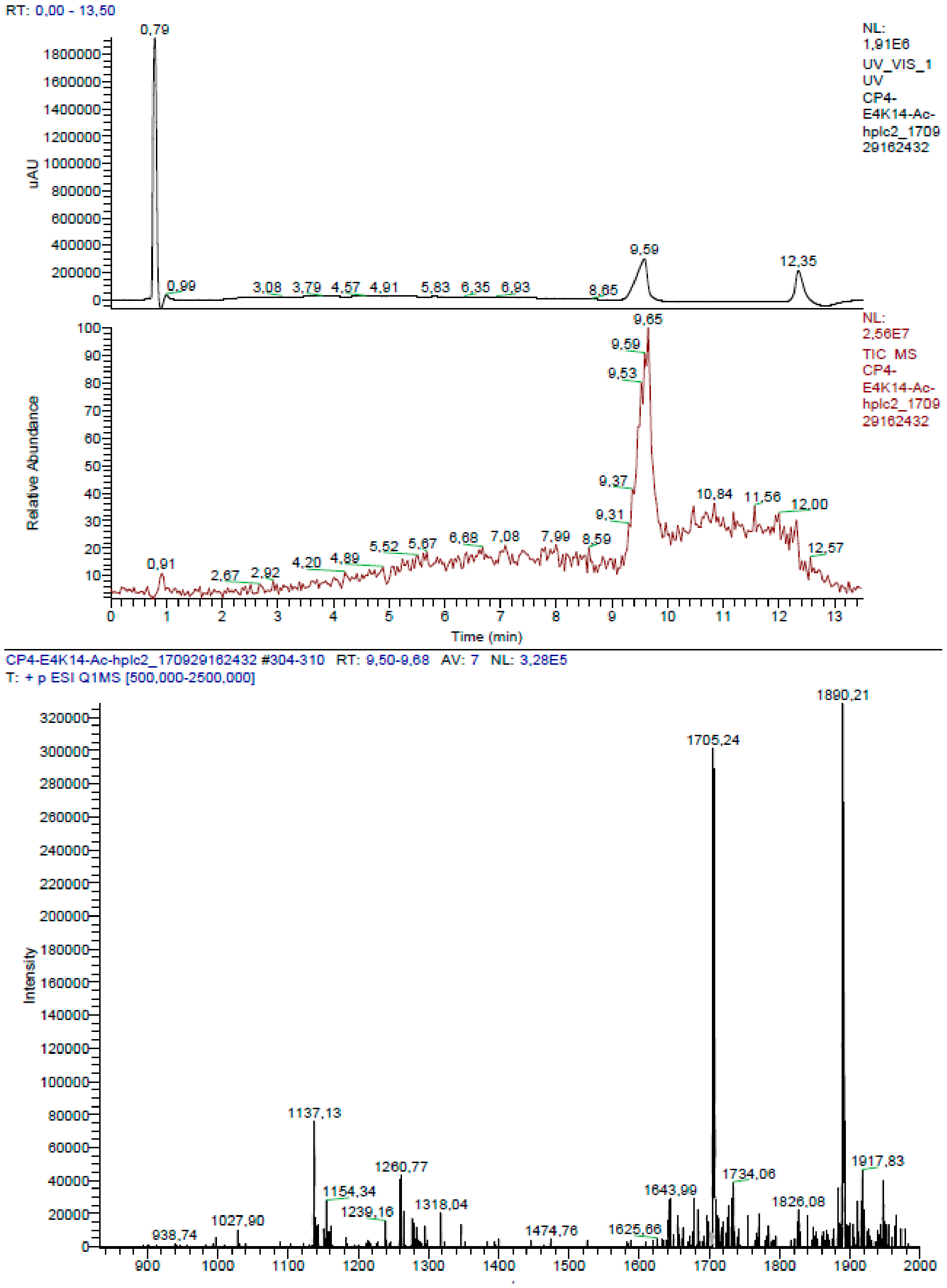

Figure S5: LCMS spectrum of purified peptide AcCPE.

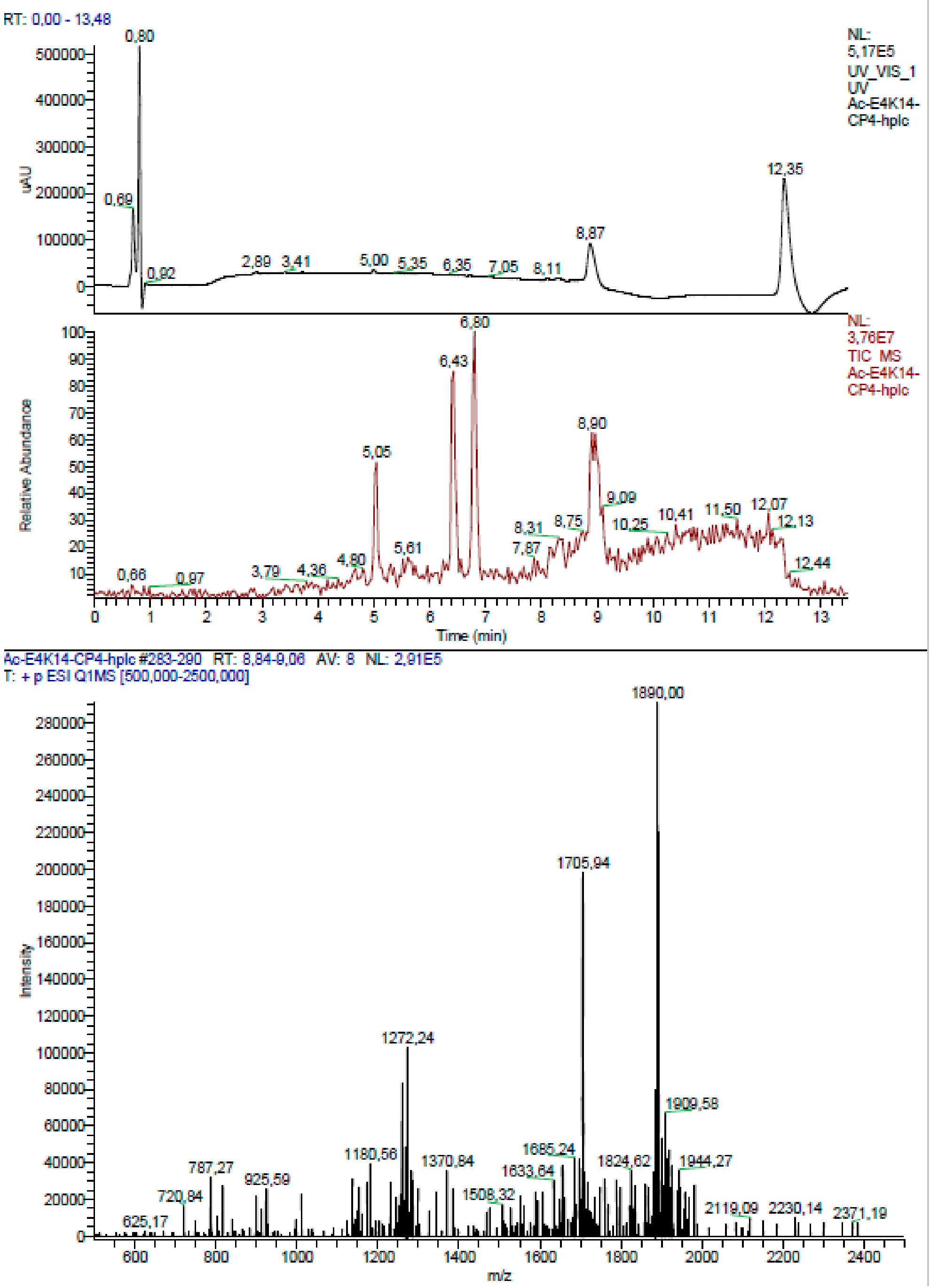

Figure S6: LCMS spectrum of purified peptide fCPE.

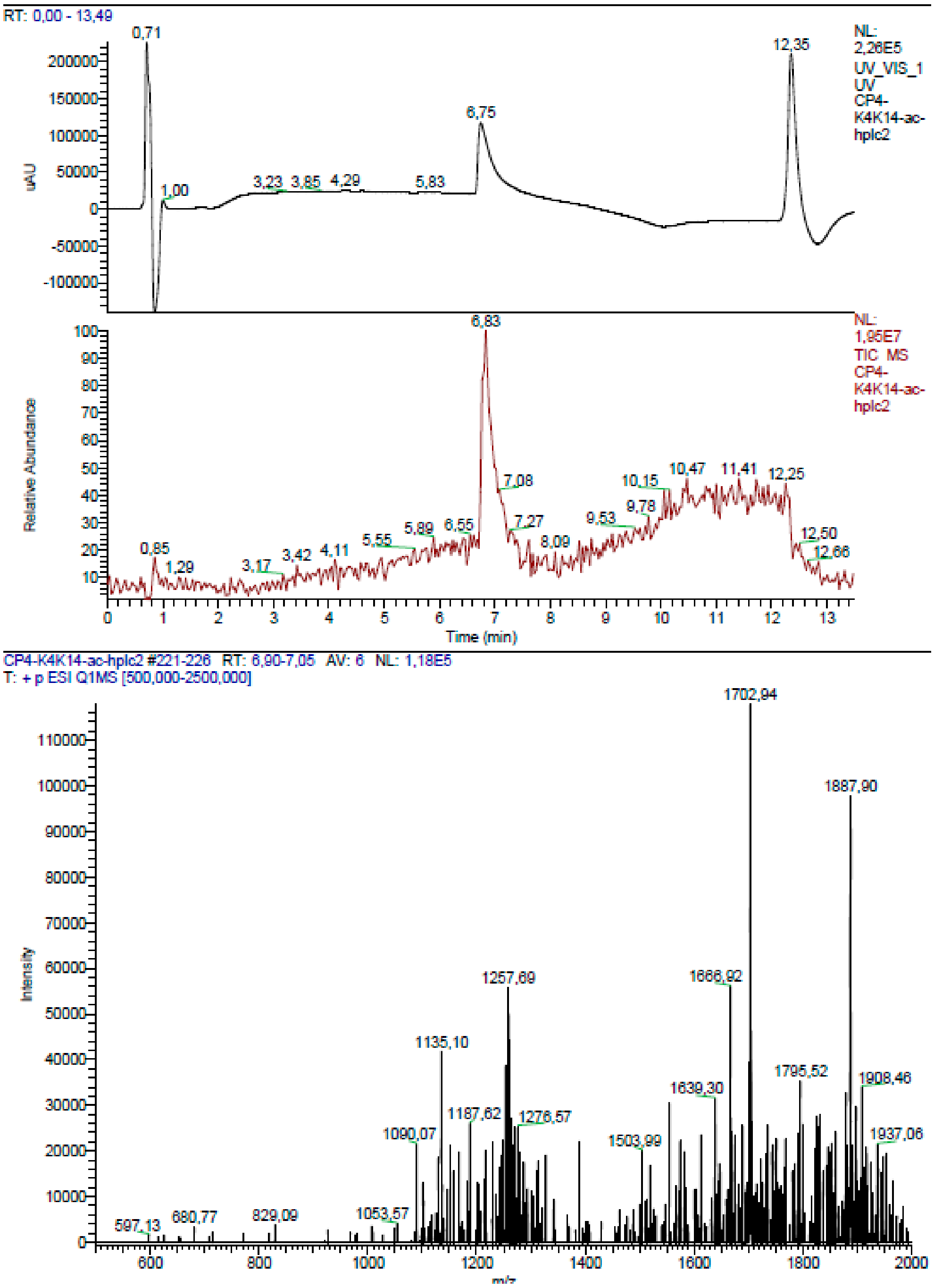

Figure S7: LCMS spectrum of purified peptide AcCPK.

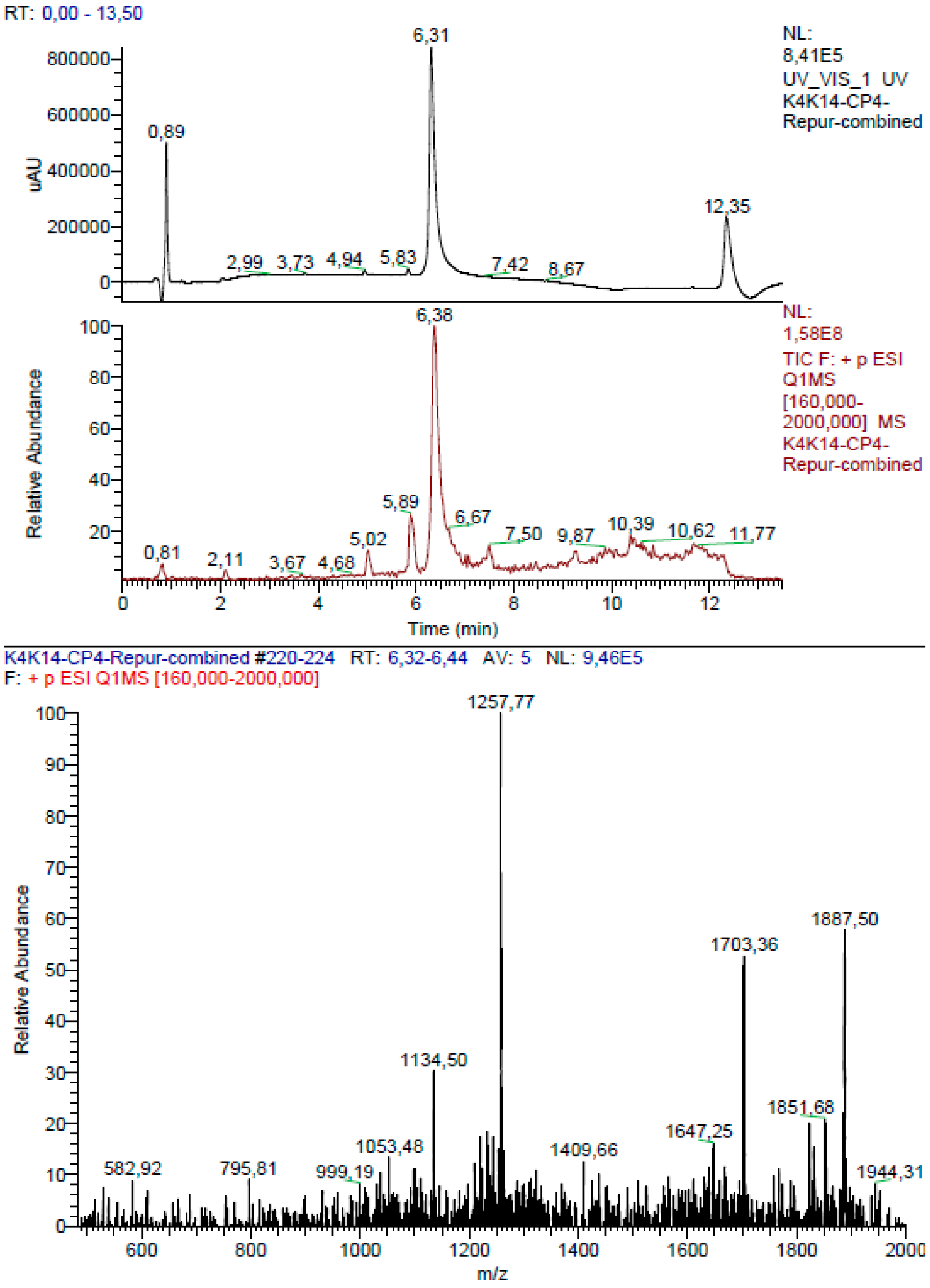

Figure S8: LCMS spectrum of purified peptide fCPK.

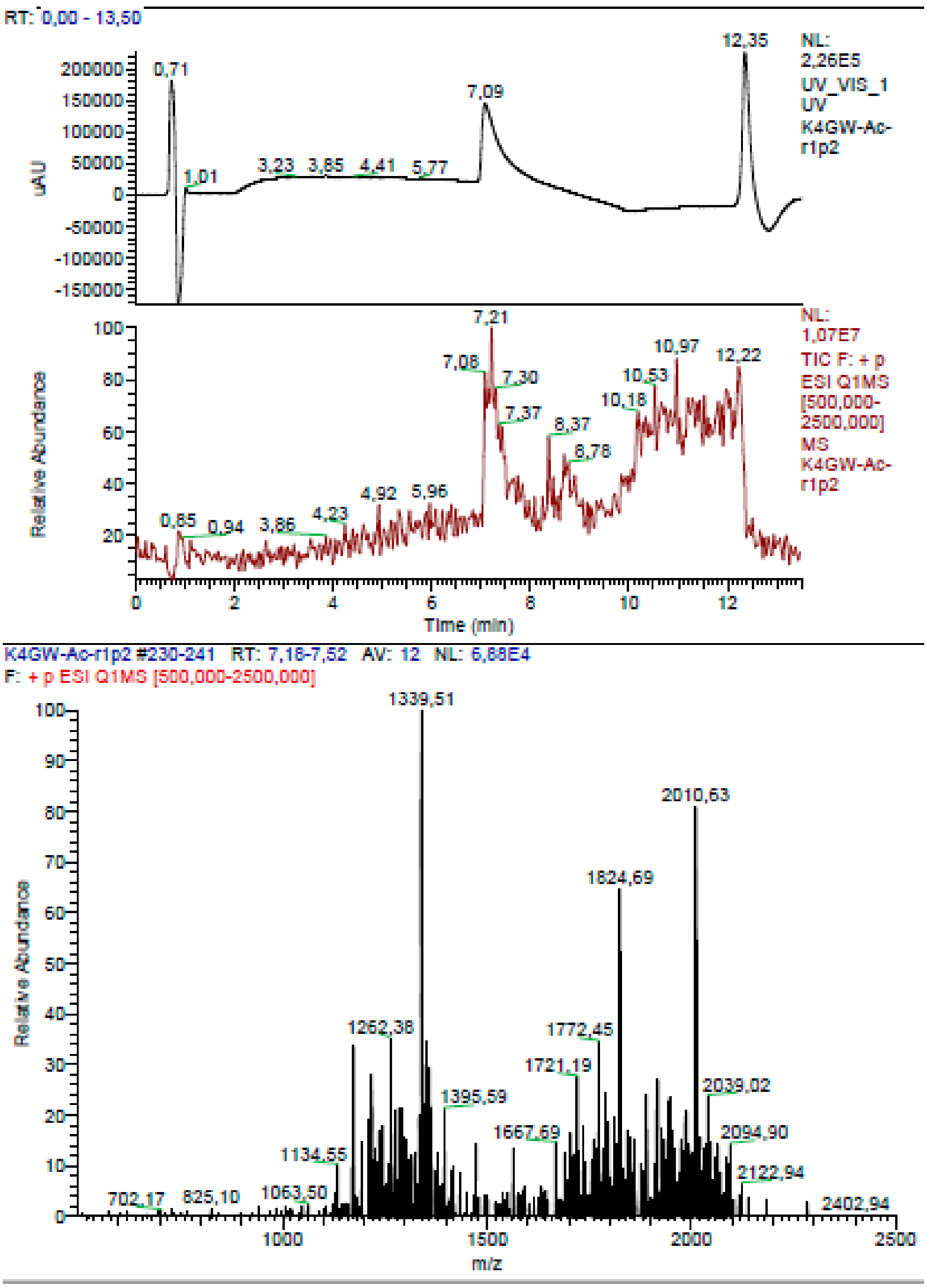

Figure S9: LCMS spectrum of purified peptide AcCPK-GW.

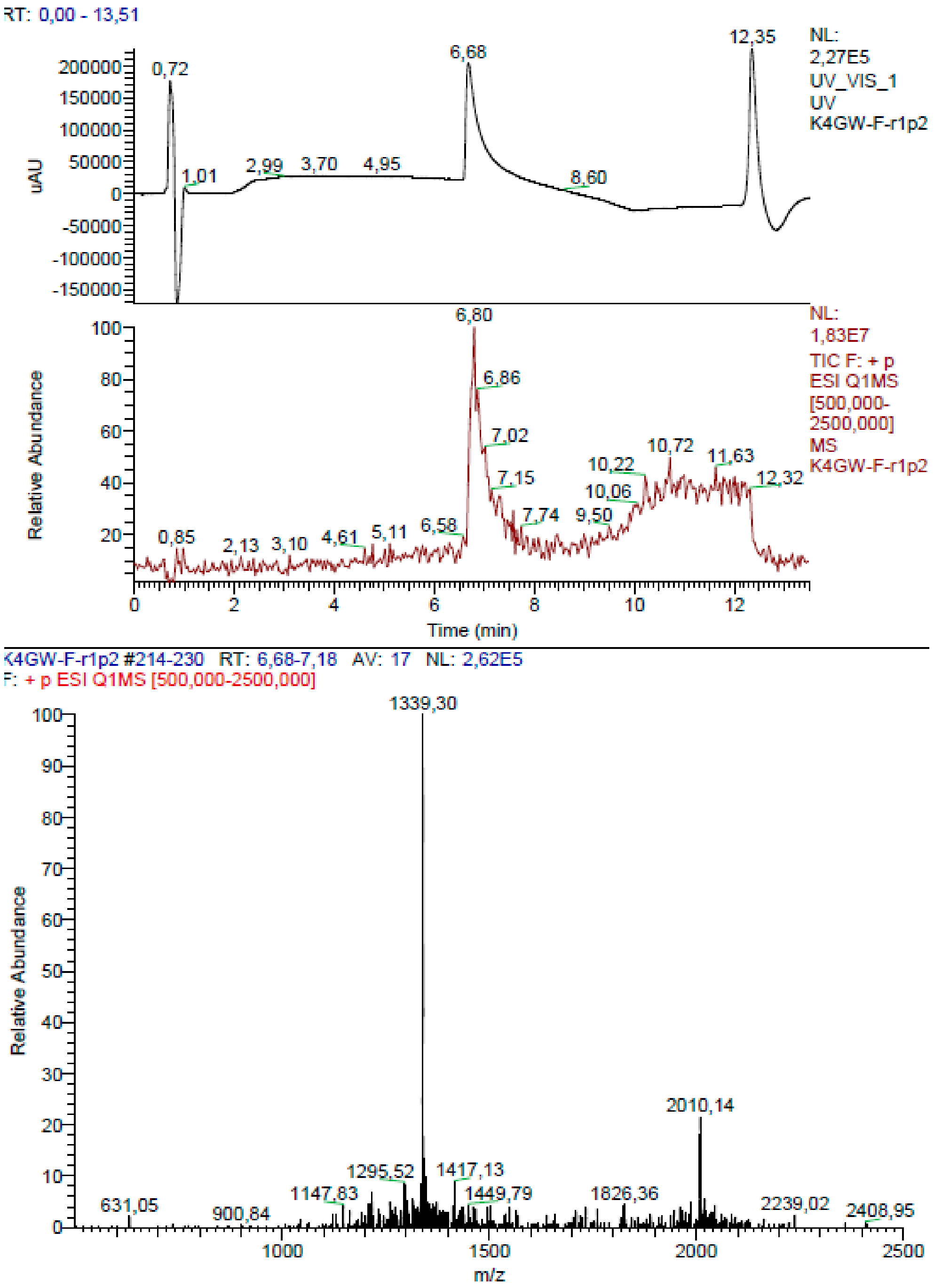

Figure S10: LCMS spectrum of purified peptide fCPK-GW

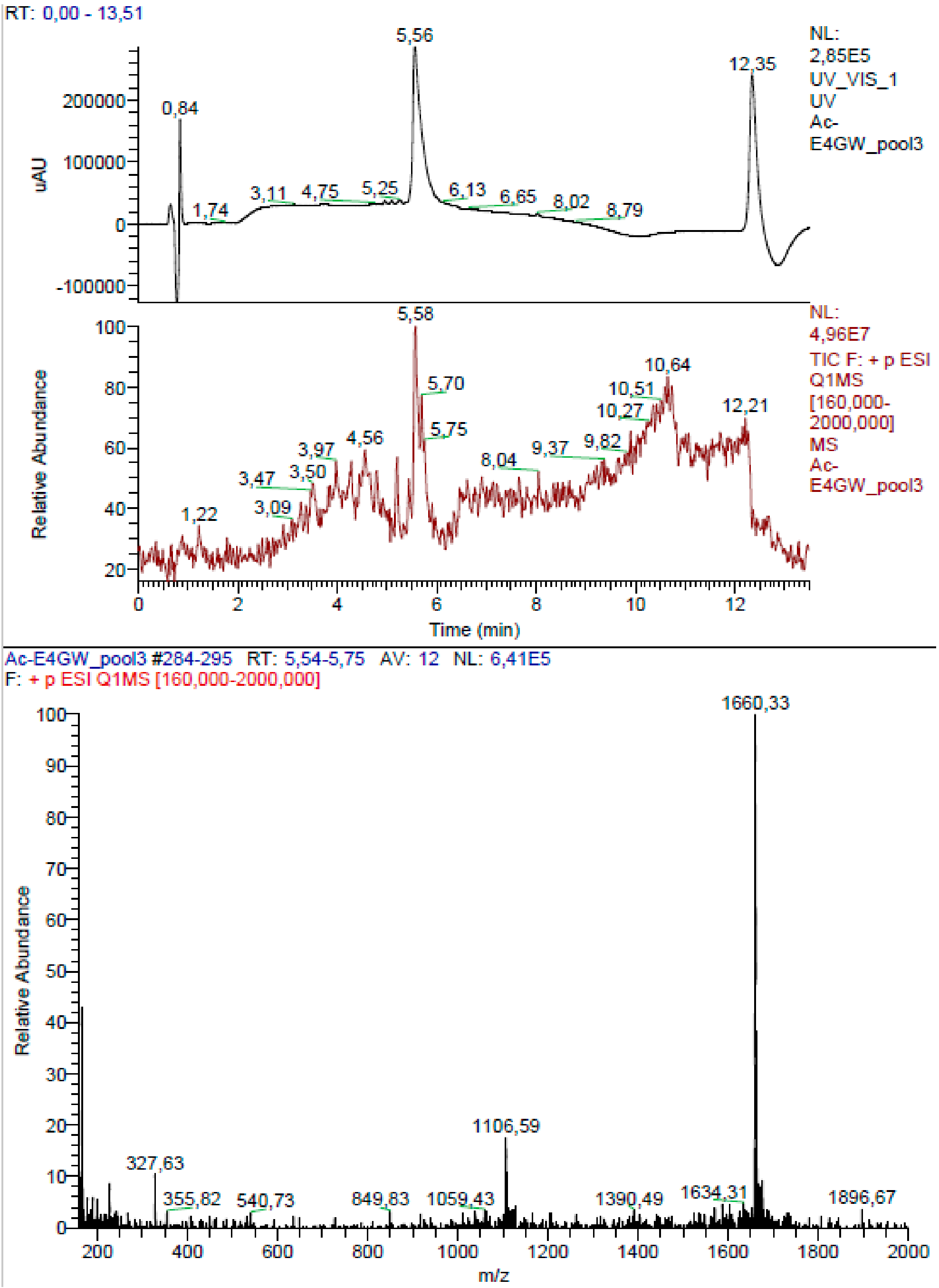

Figure S11: LCMS spectrum of purified peptide E_4GW.

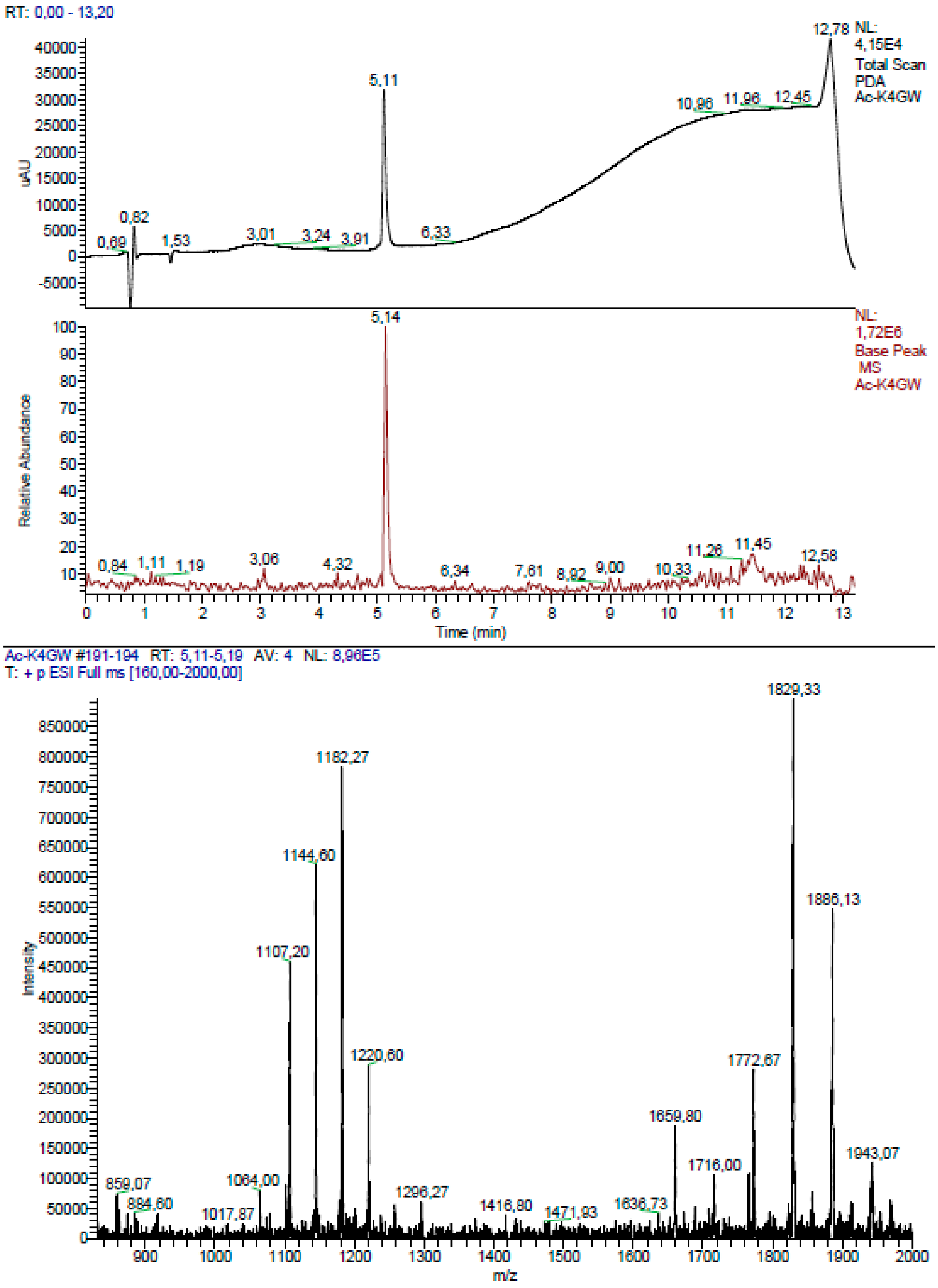

Figure S12: LCMS spectrum of purified peptide K_4GW.
